# Causal association between helicobacter pylori and atherosclerosis: a two-sample Mendelian randomization

**DOI:** 10.1186/s12872-024-03823-0

**Published:** 2024-03-15

**Authors:** Xueyi Zhang, Yue Shi, Tielin Li, Ke Chang, Yongyan Gan, Yue Feng, Xianhua Zhou

**Affiliations:** 1 Chengdu University of Traditional Chinese Medicine, Chengdu, China; 2 Meishan Shi Pengshan Qu Chinese Medicine Hospital, Meishan, China; 3 Meishan Hospital of Traditional Chinese Medicine, Meishan, China

**Keywords:** Helicobacter pylori, Atherosclerosis, CagA protein, Mendelian randomization

## Abstract

**Background:**

Helicobacter pylori (H. pylori), according to a number of recent observational studies, is connected to atherosclerosis (AS). However, the link between H. pylori and AS is debatable.

**Methods:**

In order to calculate the causal relationship between H. pylori and AS, we employed a two-sample Mendelian randomization (MR) analysis. The data for H. pylori were obtained from the IEU GWAS database (https://gwas.mrcieu.ac.uk/datasets/) and the data for AS were obtained from the Finngen GWAS database (https://r5.finngen.fi/). We selected single nucleotide polymorphisms with a threshold of 5 × 10^–6^ from earlier genome-wide association studies. MR was performed mainly using the inverse variance weighted (IVW) method. To ensure the reliability of the findings, We performed a leave-one-out sensitivity analysis to test for sensitivity. F-value was used to test weak instrument.

**Results:**

A positive causal relationship between H. pylori OMP antibody levels and peripheral atherosclerosis was shown by our two-sample MR analysis (odds ratio (OR) = 1.33, 95% confidence interval (CI) = 1.14–1.54, *P* = 0.26E-03) using IVW. Additionally, there was a causative link between coronary atherosclerosis and H. pylori VacA antibody levels (IVW OR = 1.06, 95% CI = 1.01–1.10, *P* = 0.016). All the F-values were above 10.

**Conclusions:**

This MR study discovered a causal link between H. pylori and AS. Different antibodies have different effects, so future researches are needed to figure out the exact mechanisms behind this link.

**Supplementary Information:**

The online version contains supplementary material available at 10.1186/s12872-024-03823-0.

## Background

Despite the fact Helicobacter pylori (H. pylori) is mostly innocuous and most infected people show no symptoms, infection has been connected to numerous illnesses, including peptic ulcer disease, nonulcer dyspepsia, and even cancer. The global H. pylori infection rate was 42.7% in females compared to 46.3% in males [1]. Though a asystematic review of H. pylori infection between 1980 and 2022 reported last decade the prevalence of H pylori infection declined sharply [2]. In recent years, it is widely accepted that the association between Proprotein convertase subtilisin/kexin type 9 (PCSK9) and atherosclerosis is dependent on PCSK9-mediated modulation of LDL metabolism [3]. Recent research indicates that H. pylori may also contribute to the development of atherosclerosis (AS), the most typical cardiovascular disease. According to a review infection with virulent strains of H. pylori has been linked to AS and its clinical manifestations, including myocardial infarction and ischemic stroke [4]. A systematic review and meta-analysis suggests that H. pylori infection may hasten the progression of AS, particularly in individuals under 60 years old and without cardiovascular risk factors [5]. This disorder causes massive artery lipid accumulation and inflammation, which may eventually result in myocardial infarction and stroke as its clinical complications. Clinically severe AS is a slow condition that often affects persons in their senior years. In spite of its frequency dropping in some nations, it continues to be the world's top cause of death [6].

Confounding or reverse causation are two problems that might affect observational research, Mendelian randomization (MR) can be used in epidemiology to evaluate if a risk factor causes a health issue. Gene combination randomization is similar to randomized controlled experiments.MR can improve observational study outcomes by using genetic variants as instrumental variables. Since alleles are randomly given at birth, confounding variables and reverse causality cannot affect MR. MR studies are less biased by confounding, reverse causality, and measurement errors than observational research.

There have been many clinical observational studies on the relationship between H. pylori colonization and AS, but the results are conflicting. To investigate a causal link, an MR study was conducted. Our study explores the relationship between genes and H. pylori infection. However, the GWAS of H. pylori infection was based on serological samples, which may not be truly representative of H. pylori infection. Further investigations are needed to understand the pathogenesis and provide clinical suggestions for diagnosis and treatment.

## Methods

### Study design overview

In order to measure causal effects, we used a two-sample MR approach. In short, we calculated the causative impacts of H. pylori on AS before assessing the causal influence of H. pylori on AS. Selecting acceptable genetic IVs for the associated exposure, calculating the impact size using the ivw technique, and analyzing pleiotropic effect as well as heterogeneity and sensitivity analyses are the three essential stages the study includes to verify each inference direction.

### Data sources

We acquired all SNPs and their associated summary statistics from studies that concentrate purely on European ancestry to prevent the effects of population stratification. Downloadable GWAS summary statistics for H. pylori, including Anti-H. pylori IgG, H. pylori VacA antibody levels (CagA), H. pylori OMP antibody levels Catalase, H. pylori OMP antibody levels (OMP), and VacA, were available in the Ebi GWAS database (https://gwas.mrcieu.ac.uk/datasets/). There were 15,489 participants involved in the H. pylori study [7] and everyone who took part was of European descent. The AS was diagnosed in the standard of ICD-10—I70. It was possible to download the GWAS summary statistics for AS, including cerebral atherosclerosis (Cereb.AS) (150 cases, 241202 controls), coronary atherosclerosis (Coron.AS) (28598 cases, 222551 controls), peripheral atherosclerosis (PAD) (8393 cases, 190836 controls), and other AS (8391 cases, 244907 controls), from the Finngen GWAS database (https://r5.finngen.fi/).

### Instrumental variable selection

As instrumental variables (IVs) in MR, genetic variants are exploited. The researchers use two distinct populations to examine the effect of IVs on exposures and outcomes, using openly available data from in-depth GWAS to inform their research. To obtain unbiased results, a number of conditions must be satisfied, including (a) that the IVs have a statistically significant relationship to the exposure; (b) that the IVs are not linked to confounders that are related to the exposure or the outcome, particularly the outcome; and (c) that the IVs only affect the outcome through exposure. Based on the three MR assumptions, the SNPs were chosen as IVs.

To ascertain the first hypothesis, we first selected statistically significant single-nucleotide polymorphisms (SNPs) below the genome-wide statistical significance threshold (5 × 10^–8^) to serve as IVs. Unfortunately, only a small number of gut microbiota were selected as IVs after selecting SNPs. To explore more relations between H. pylori and atherosclerosis and obtain more comprehensive results, we used the second threshold that identified SNPs smaller than the locus-wide significance level (5 × 10^−6^) [8] and selected them as the second IVs set to find more potential causal associations [9, 10]. Additionally, these SNPs showed a substantial degree of mutational potential (minor allele frequency; MAF > 5%). To remove SNPs linked to considerable linkage disequilibrium (LD), we utilized a clumping approach with *R*^2^ < 0.001 and a window size of 10,000 kb.

Additionally, we calculated the cumulative F statistics for SNPs using the equation below: F = (beta/se)^2^. The F statistics presented in the table are average F statistics. F-statistics were used to evaluate the efficacy of the instrument (a device with an F value < 10 was considered weak).

### MR analysis

All the analyses were performed in R version 4.2.2 with the packages Two Sample MR (version 0.5.6). In our main study, the inverse variance weighted (IVW) method, MR-Egger method and Weighted median were used to assess the causal associations. When results were inconsistent through these methods, we prefered to use the IVW technique which has the best statistical validity and consistently calculates the causal impact of exposure on the outcome [11]. The method's ability to minimize volatility is a benefit that could lead to more accurate results when measuring SNP-exposure effects with measurement error [12]. When all IVs were legitimate, it delivered a more accurate risk estimate than other techniques [13].

The relative risk associated with the designated disease was determined using the odds ratio (OR) and 95% confidence interval (CI). For quality control, pleiotropy, heterogeneity, and sensitivity analyses were utilized. To determine whether horizontal pleiotropy is present, we employed the MR-Egger regression. Additionally, pleiotropy was examined using the MR-PRESSO test, and any SNPs that were outliers were manually removed. Additionally, outliers were found and eliminated using radial MR. Heterogeneity was evaluated using the IVW approach and egger regression, and it was quantified using Cochran's Q statistic [14]. In order to test the reliability of the results, the leave-one-out method recalculates the results after removing each individual SNP one at a time. We eliminated particular SNPs that had an adverse impact on the findings.

## Results

**Fig. 1:**
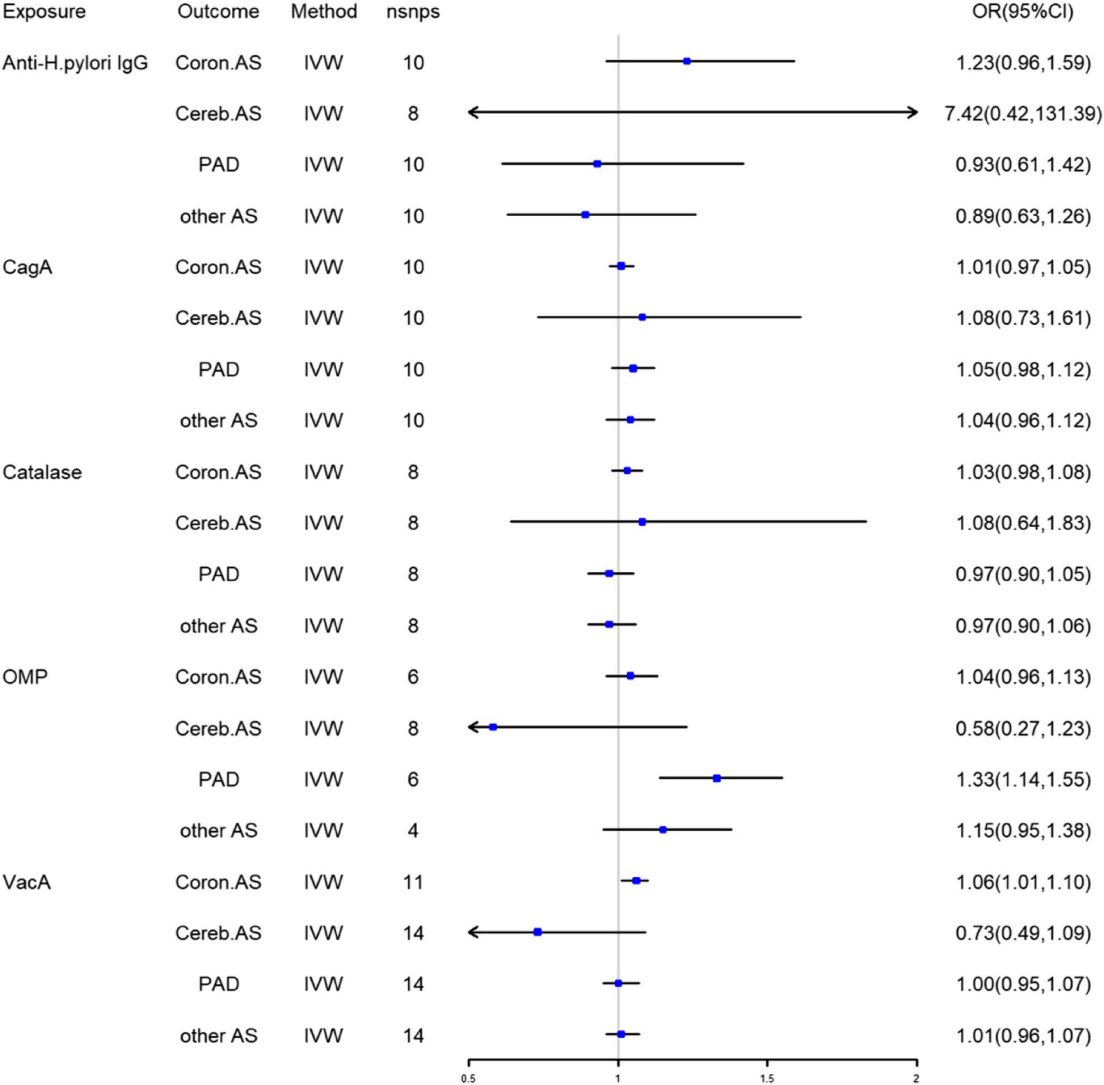
Causal estimates given as odds ratios, 95% confidence intervals and its sub-types

Four to fourteen SNPs for H. pylori were included after strict exclusion criteria. All F-statistics were above 10, indicating that there was no instrument variable bias. The MR estimations using the IVW technique were shown in Fig. 1. OMP and PAD were found to be positively causally related (IVW OR = 1.33, 95% CI = 1.14–1.54, *P* = 0.26E-03). There was a causative link between Coron.AS and VacA (IVW OR = 1.06, 95% CI = 1.01–1.10, *P* = 0.016). Anti-H. pylori IgG, Catalase, and VacA were genetically predicted by the IVW to have no causal effect on AS (IVW OR = 1,03, 95% CI = 0,82–1,28, *P* > 0.05).

Table 1 showed that the results of heterogeneity test and pleiotropy test were gather than 0.05, suggesting that there was no heterogeneity or multiplicity. In addition, the Supplementary Table 1 provides OR and 95% CI for all associations to give the reader a clearer picture of the strength and precision of the observed relationships.

**Table 1:** The result of heterogeneity test and pleiotropy test of MR analysis

exposures	outcomes	nSNPs	Heterogeneity test			Pleiotropy test	F
			method	Q	P		
Anti-H.pylori IgG	Other AS	10	MR-Egger IVW	8 9	0.05 0.06	0.54	22.92
	Cereb.AS	8	MR-Egger IVW	6 7	0.90 0.91	0.50	22.98
	Coron.AS	10	MR-Egger IVW	8 9	0.25 0.25	0.37	22.92
	PAD	10	MR-Egger IVW	8 9	0.31 0.24	0.20	22.92
CagA	Other AS	10	MR-Egger IVW	8 9	0.05 0.06	0.54	22.49
	Cereb.AS	10	MR-Egger IVW	8 9	0.46 0.53	0.58	22.49
	Coron.AS	10	MR-Egger IVW	8 9	0.70 0.78	0.83	22.49
	PAD	10	MR-Egger IVW	8 9	0.13 0.18	0.80	22.49
Catalase	Other As	8	MR-Egger IVW	6 7	0.18 0.22	0.54	22.91
	Cereb.AS	8	MR-Egger IVW	6 7	0.74 0.71	0.34	22.91
	Coron.AS	8	MR-Egger IVW	6 7	0.70 0.79	0.84	22.91
	PAD	8	MR-Egger IVW	6 7	0.42 0.53	0.84	22.91
OMP	Other AS	4	MR-Egger IVW	2 3	0.82 0.73	0.44	22.32
	Cereb.AS	9	MR-Egger IVW	6 7	0.90 0.91	0.50	24.40
	Coron.AS	8	MR-Egger IVW	4 5	0.25 0.25	0.37	24.20
	PAD	6	MR-Egger IVW	4 5	0.47 0.27	0.17	23.23
VacA	Other AS	14	MR-Egger IVW	12 13	0.93 0.89	0.25	23.19
	Cereb.AS	14	MR-Egger IVW	12 13	0.81 0.79	0.32	23.19
	Coron.AS	12	MR-Egger IVW	9 10	0.70 0.73	0.44	22.87
	PAD	13	MR-Egger IVW	12 13	0.81 0.85	0.64	23.13

*Anti-H.pylori IgG* Anti-H. pylori IgG levels, *CagA* H. pylori CagA antibody levels, *Catalase* H. pylori Catalase antibody levels, *OMP* H. pylori OMP antibody levels, *VacA* H. pylori VacA antibody levels, *Other AS* Atherosclerosis, excluding cerebral, coronary and PAD, *Cereb.AS* Cerebral atherosclerosis, *Coron.AS* Coronary atherosclerosis, *PAD* Peripheral atherosclerosis

The forest plot depict of causal correlations between genetically predicted H. pylori and AS (Supplementary Figure 1–5). And we performed a Leave-one-out sensitivity analysis to test for sensitivity. We removed outliers of SNPs that had a subversively substantial impact on the results for the robustness (Supplementary Figure 6–10).

## Discussion

H. pylori and AS are causally related, according to our MR investigation. OMP and PAD have a causal relationship, and VacA and Coron.AS have a causal relationship.

A routine medical exam revealed a connection between the onset of carotid plaque and the presence of H. pylori [15]. According to the data, young Chinese males under the age of fifty may be at risk for carotid AS if they have H. pylori infection [16]. According to a recent study published in 2022, H. pylori infection may put women under the age of sixty at risk of developing cerebral AS [17]. H. pylori infection can considerably increase Carotid intima-media thickness (CIMT), according to a meta-analysis [5]. According to a comprehensive survey conducted in Iran in 2022, there was a strong correlation between this bacterium infection and a twofold increase in the risk of AS in that country's population (OR: 1.44; 95% CI: 1.07–1.95) [18]. We established a link between an H. pylori infection and AS. Despite the fact that several studies have shown a connection between H. pylori and AS, some investigations have produced contradictory findings [19]. Gastroenterological guidelines recommend eradication of H. pylori in patients with manifest atherosclerosis. H. pylori did not appear to contribute to carotid AS, according to a study of Japanese patients having carotid endarterectomy surgery [20]. According to a cross-sectional investigation of elderly dyspeptic individuals, H. pylori infection was not linked to AS [21]. A study based on Mendelian randomization confirm that the causal effect of H. pylori infection on coronary heart disease (CHD) incidence is mediated by BMI [22] while another latest study thought that H. pylori positivity and CV risk were independently associated however the difference did not translate into CVD mortality between patients with and without the bacteria in a cohort [23]. Hence, conflicting data exist regarding the relationship between H. pylori and atherosclerosis and further investigations are needed in order to elucidate this relationship [23].

Numerous other research have concentrated on the processes between H. pylori with AS. The mechanisms proposed for a causal link between H. pylori infection and AS include arterial stiffness, elevated blood pressure, malnutrition, intensified inflammatory stress, dyslipidemia, abnormal glucose metabolism, and dyslipidemia. Results from other studies, however, suggested that there may not be a causal relationship between H. pylori infection and AS and that common risk factors, such as aging, smoking, having a poor socioeconomic status, and eating a lot of salt, may significantly contribute to their coexistence in the population [24].

H.pylori and AS have not been studied before using an MR design. The precise mechanisms by which H. pylori raises the risk of AS remain unclear. AS is originally believed to be a straightforward lipid accumulation lesion, a MR [25] revealed the causality of anti-H. pylori IgG levels on HDL cholesterol levels which are associated with increased risks of Myocardial Infarction. But in recent years, new research has revealed that AS is actually a chronic inflammatory disease, showing that persistent infection is crucial for the growth of AS. It is not unexpected that in some circumstances inflammatory disorders like AS are induced by incorrect activation of them because they play such a crucial role in starting immune responses to a range of pathogens.

TLRs are considered to play an important part in the progression of AS. The dysfunction of vascular cells, the recruitment of macrophages and other immune cells to the site of vascular injury, the generation of foam cells, and the instability of plaques are a few of the mechanisms of atherogenesis induced by TLRs [15].

H. pylori releases vesicles that are fragments of its outer membrane. These H. pylori OMVs include a wide variety of bacterial antigens and virulence factors. These processes involve, at least in part, CagA and LPS from H. pylori-derived OMVs. Various scientific studies have been conducted on CagA, which is produced by H. pylori-infected gastric epithelial cells, promotes the production of atherosclerotic foam cells and macrophage-derived foam cells. Through the NLRP3-IL1 signaling pathway, The CagA protein produced by H. pylori can increase aortic EC adhesion and promote AS. LPS from H. pylori stimulates the NF-B pathway, which in turn activates the IL-8 pathway through TLR4 ligation [19]. These provide a unique pathway between AS and H. pylori infection.

The blood-group antigen-binding genes (babA, babB, and alpAB) and adherence-associated lipoprotein (alpAB), which all belong to the family of large outer membrane proteins found in H pylori, are primarily involved in adherence. H. pylori can affect signal transduction pathways due to its close proximity to the gastric epithelium, which causes proinflammatory cytokines like interleukin-8 (IL8) to be produced by epithelial cells [7]. It has been demonstrated that after H. pylori infection, the outer inflammatory protein, a member of the large H. pylori OMP family, contributes to the activation of IL-8 in epithelial cells [26]. The presence of the cag pathogenicity island was necessary for interleukin-8 (IL-8) expression in gastric cells, and the production of BabA and OipA was positively linked with the presence of the translocated effector protein CagA [26].

Omp may therefore increase the risk of AS by increasing the adhesion of aortic ECs, causing the production of proinflammatory cytokines like interleukin-8 (IL8), which is dependent on the effector protein CagA, and by boosting the NLRP3-IL1 signaling pathway. It is undeniable that H. pylori raises the chance of acquiring AS. The link between H. pylori and AS may help with prevention for AS patients as well as better AS treatment. The exact mechanistic pathways connecting H. pylori to AS need more detailed exploration and evidence. Similarly, further studies are needed to explore and test whether interventions to improve.

Chronic inflammation plays a major role in the pathogenesis of atherosclerosis. Vascular smooth muscle cells (VSMC), by expressing and presenting major histocompatibility complex II (MHC II) molecules, help to recruit T lymphocytes and initiate the inflammatory response within the vasculature. We find that one SNP is at the CIITA Gene, which is considered the master general regulator of MHC II transcription, indicating that H. pylori and AS share a common gene [27].

The use of MR, which can reflect causal factors distinct from short-term self-reported H.pylori infection or lifetime exposure to H.pylori, was the primary strength of the present study. To the best of our knowledge, our study is the first MR analysis of this topic. We exerted multiple endeavors to satisfy MR's fundamental presumptions. The study utilized a large sample size and SNPs from GWAS to establish statistically valid causation. These measurements contribute to the validation of the findings.

Our efforts are subject to some restrictions. First of all, the dataset we used exclusively contained people from Europe. Although population stratification bias can be reduced by utilizing just one European community to study the causal relationship, which may lead to bias estimates and it might not be transferable to other populations. Second, few SNPs were below the common bioinformatic cutoff of p < 5 × 10^−8^. This quantity of SNPs could diminish any connections while also making it challenging to match IVs in the results. Therefore, we chose SNPs with a less strict significance of 5 × 10^−6^. Previous research have recommended this strategy [28] with the restriction that it might result in a slight bias in instrumental variables. Finally, due to the limitations of MR in establishing causality between H. pylori and AS, clues and evidence from multiple observational and experimental studies can be combined to strengthen causal inference [29]. There may be an influence from unmeasured confounders.

In conclusion, this study uses a Mendelian randomization study to examine the causal association between H. pylori colonization and the risk of AS. This research sheds important light on how AS develops and possible risk factors by examining the genetic foundation of the disease. The findings of this study may serve as a foundation for future research on the prevention and treatment of AS and will aid in our understanding of how H. pylori influences AS development.

### Outcomes

Our study is the first to use a bidirectional MR analysis to demonstrate the causal link between H. pylori infection and an elevated risk of developing AS. The research may help systems biologists better understand the pathogenic causes of AS. Our study does have certain drawbacks, though. In the GWAS data, serological testing may have altered the diagnosis of H. pylori infection, was used to make the diagnosis of H. pylori infection. Second, the population of Europe was the only one included in the dataset we used. First and foremost, our research served as a reminder to doctors that when H. pylori infection is identified, precautions and coordinated efforts for AS prevention and early management should be taken into account.

### Supplementary Information

**Supplementary Material 1.**

## Data Availability

The original data for this study are available from publicly available databases (https://gwas.mrcieu.ac.uk/datasets/, https://r5.finngen.fi/). The present study did not need ethics approval, further enquiries can be directed to the corresponding author.
