# Supplementary material for: Causal association between helicobacter pylori and atherosclerosis: a two-sample Mendelian randomization
Source: BMC Cardiovasc Disord. 2024 Mar 15;24:161. doi: 10.1186/s12872-024-03823-0 (PMC10941435; doi:10.1186/s12872-024-03823-0)
Supplement: Supplementary file 1 — Supplementary Material 1. [file 12872_2024_3823_MOESM1_ESM.docx]

**Supplementary Material**

Supplyment Table 1. Mendelian Randomization analysis of the causal association between helicobacter pylori and atherosclerosis.

| exposures | outcomes | nSNPs | methods | *P* | OR | 95% CI |
| --- | --- | --- | --- | --- | --- | --- |
| Anti-H.pylori IgG | Other AS | 10 | MR-Egger  Weighted median  Ivw | 0.15  0.86  0.51 | 2.74  0.96  0.89 | 0.80-9.37  0.59-1.56  0.63-1.26 |
|  | Cereb.AS | 8 | MR-Egger  Weighted median  Ivw | 0.32  0.47  0.17 | 234.63  3.70  7.4 | 0.01-14961792.68  0.11-124.46  0.42-131.39 |
|  | Coron.AS | 10 | MR-Egger  Weighted median  Ivw | 0.67  0.14  0.10 | 0.81  1.27  1.23 | 0.33-2.01  0.92-1.74  0.96-1.59 |
|  | PAD | 10 | MR-Egger  Weighted median  Ivw | 0.25  0.66  0.75 | 2.43  0.89  0.93 | 0.60-9.91  0.52-1.51  0.61-1.42 |
| CagA | Other AS | 10 | MR-Egger  Weighted median  Ivw | 0.91  0.54  0.32 | 0.99  1.03  1.04 | 0.84-1.17  0.94-1.11  0.96-1.12 |
|  | Cereb.AS | 10 | MR-Egger  Weighted median  Ivw | 0.75  0.24  0.70 | 0.87  1.39  1.08 | 0.37-2.04  0.80-2.41  0.73-1.61 |
|  | Coron.AS | 10 | MR-Egger  Weighted median  Ivw | 0.95  0.45  0.58 | 1.00  1.02  1.01 | 0.93-1.08  0.97-1.07  0.97-1.05 |
|  | PAD | 10 | MR-Egger  Weighted median  Ivw | 0.74  0.99  0.20 | 1.03  1.00  1.05 | 0.88-1.20  0.92-1.09  0.98-1.12 |
| Catalase | Other AS | 8 | MR-Egger  Weighted median  Ivw | 0.83  0.84  0.54 | 1.02  0.99  0.98 | 0.87-1.20  0.90-1.09  0.90-1.06 |
|  | Cereb.AS | 8 | MR-Egger  Weighted median  Ivw | 0.34  0.72  0.77 | 1.67  1.14  1.08 | 0.63-4.43  0.56-2.31  0.64-1.82 |
|  | Coron.AS | 8 | MR-Egger  Weighted median  Ivw | 00.45  0.18  0.25 | 1.03  1.04  1.03 | 0.95-1.13  0.98-1.11  0.98-1.07 |
|  | PAD | 8 | MR-Egger  Weighted median  Ivw | 0.84  0.57  0.48 | 0.99  0.97  0.97 | 0.86-1.13  0.87-1.08  0.90-1.05 |
| OMP | Other AS | 4 | MR-Egger  Weighted median  Ivw | 0.28  0.46  0.14 | 1.41  1.09  1.15 | 0.89-2.23  0.86-1.37  0.95-1.38 |
|  | Cereb.AS | 9 | MR-Egger  Weighted median  Ivw | 0.90  0.06  0.16 | 2.32  0.02  0.21 | 0.07-0.85  0.16-0.40  0.27-.0.58 |
|  | Coron.AS | 8 | MR-Egger  Weighted median  Ivw | 0.64  0.18  0.33 | 1.07  1.07  1.04 | 0.82-1.38  0.97-1.20  0.96-1.13 |
|  | PAD | 6 | MR-Egger  Weighted median  Ivw | 0.05  0.04  0.00 | 1.11  0.41  0.44 | 1.22-1.93  1.01-1.23  1.14-1.32 |
| VacA | Other AS | 14 | MR-Egger  Weighted median  Ivw | 0.41  0.41  0.72 | 0.06  0.11  0.06 | 0.86-0.95  0.96-1.03  0.96-1.01 |
|  | Cereb.AS | 14 | MR-Egger  Weighted median  Ivw | 0.92  0.29  0.13 | 1.04  0.75  0.73 | 0.47-2.27  0.44-1.28  0.49-1.09 |
|  | Coron.AS | 12 | MR-Egger  Weighted median  Ivw | 0.11  0.27  0.02 | 0.19  0.10  0.10 | 0.99-1.10  0.97-1.04  1.01-1.05 |
|  | PAD | 13 | MR-Egger  Weighted median  Ivw | 0.75  0.58  0.03 | 0.10  0.11  0.06 | 0.87-0.98  0.84-1.02  0.95-1.00 |

Anti-H.pylori IgG, Anti-H. pylori IgG levels. CagA, H. pylori CagA antibody levels. Catalase, H. pylori Catalase antibody levels. OMP ,H. pylori OMP antibody levels. VacA, H. pylori VacA antibody levels. Other AS, Atherosclerosis, excluding cerebral, coronary and PAD. Cereb.AS, Cerebral atherosclerosis. Coron.AS, Coronary atherosclerosis. PAD, Peripheral atherosclerosis.


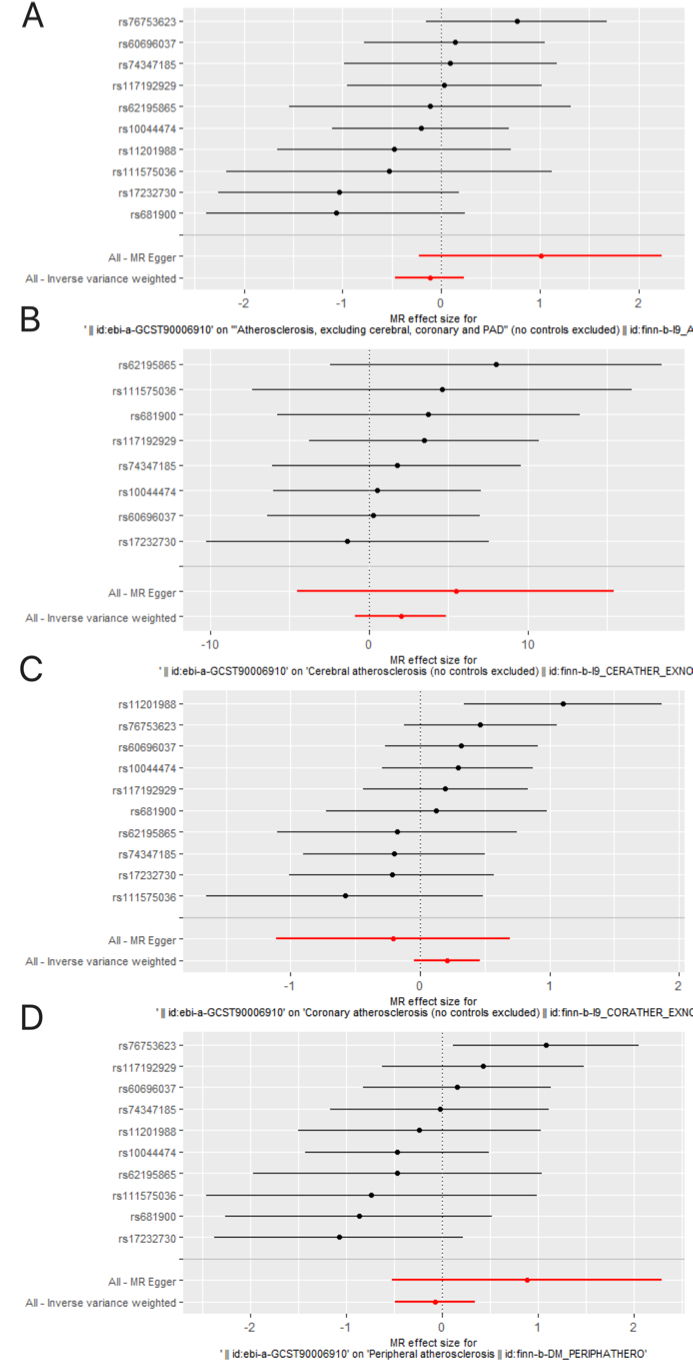


Supplementary Figure1. Forest plots of anti-helicobacter pylori IgG on atherosclerosis. (A) Atherosclerosis; (B) Cerebral atherosclerosis; (C) Coronary atherosclerosis;(D) Peripheral atherosclerosis.


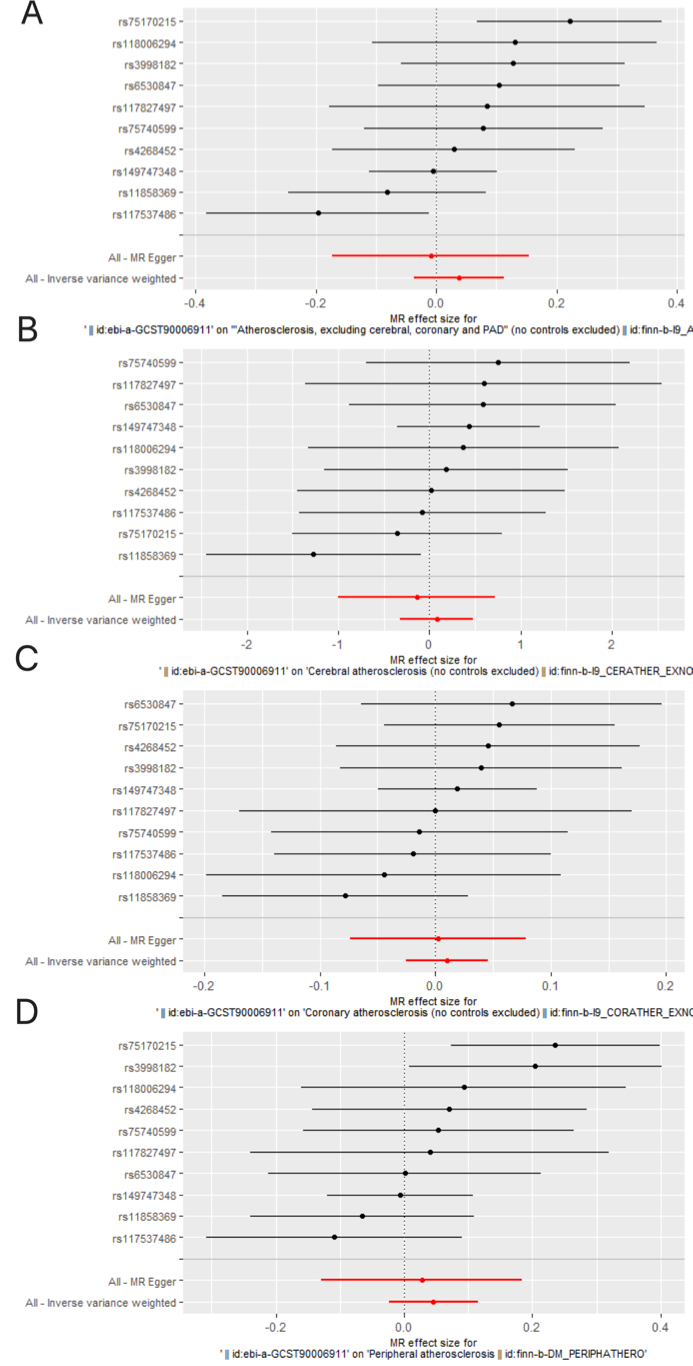


Supplementary Figure 2. Forest plots of helicobacter pylori CagA antibody on atherosclerosis. (A) Atherosclerosis; (B) Cerebral atherosclerosis; (C) Coronary atherosclerosis;(D) Peripheral atherosclerosis;


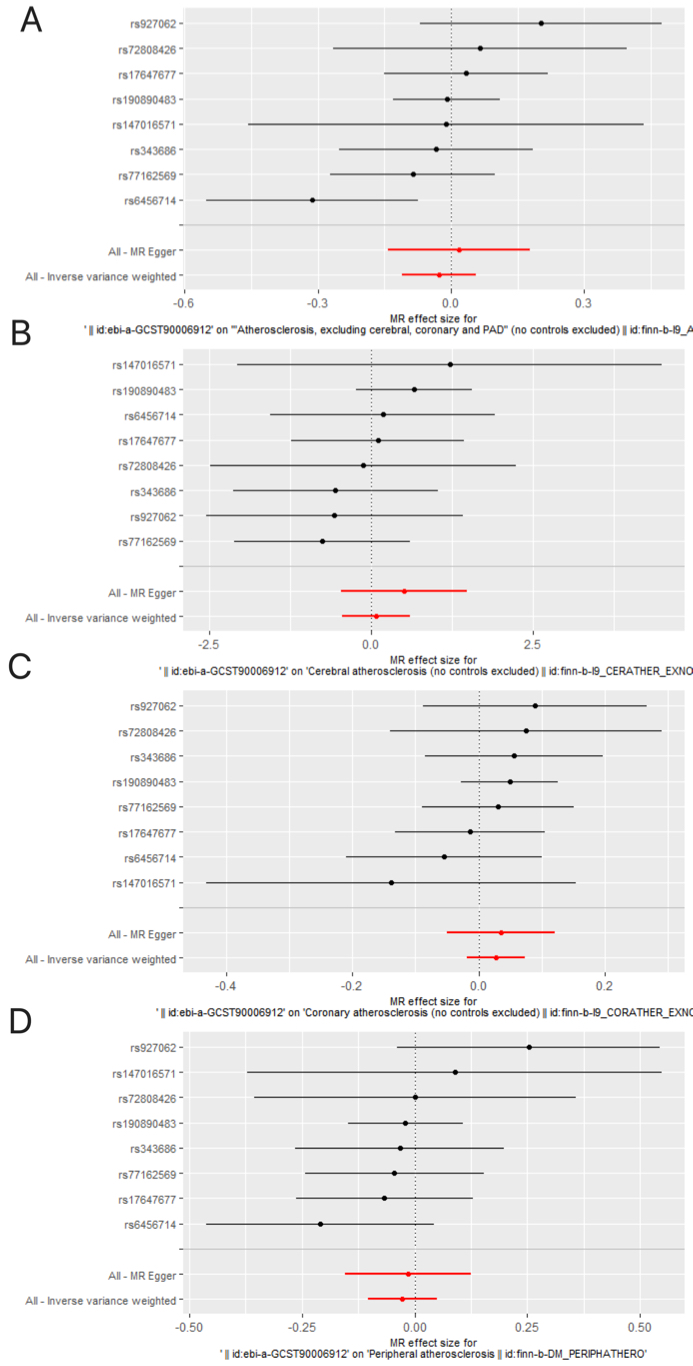


Supplementary Figure 3. Forest plots of helicobacter pylori Catalase antibody on atherosclerosis. (A) Atherosclerosis; (B) Cerebral atherosclerosis; (C) Coronary atherosclerosis;(D) Peripheral atherosclerosis;


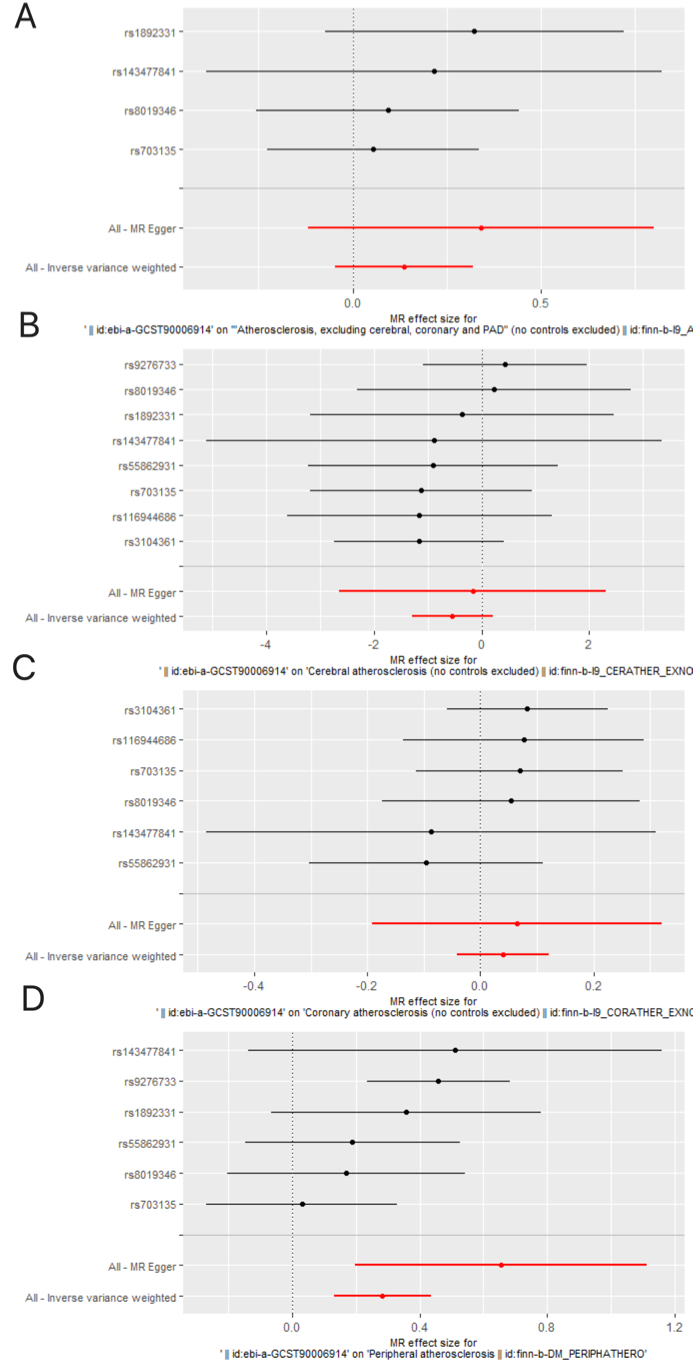


Supplementary Figure 4. Forest plots of helicobacter pylori OMP antibody on atherosclerosis. (A) Atherosclerosis; (B) Cerebral atherosclerosis; (C) Coronary atherosclerosis;(D) Peripheral atherosclerosis;


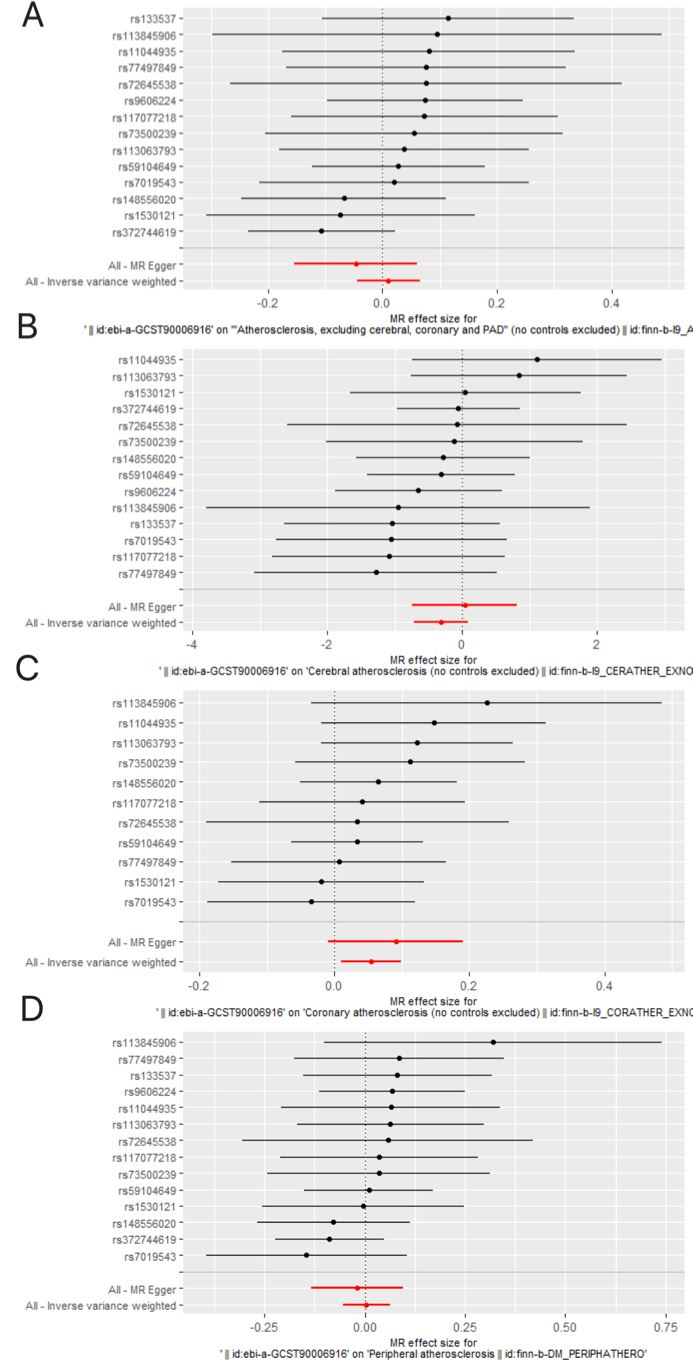


Supplementary Figure 5. Forest plots of helicobacter pylori VacA antibody on atherosclerosis. (A) Atherosclerosis; (B) Cerebral atherosclerosis; (C) Coronary atherosclerosis;(D) Peripheral atherosclerosis;


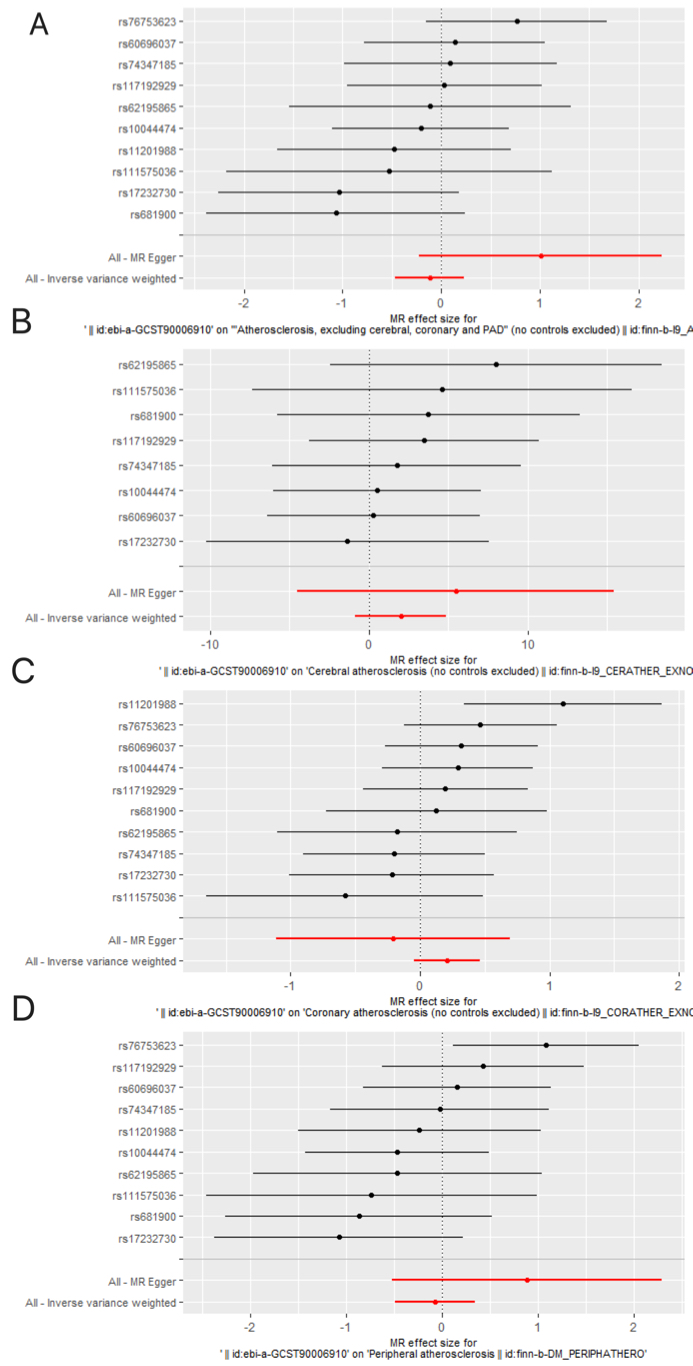


Supplementary Figure 6. Leave one out of sensitivity tests of anti-helicobacter pylori IgG on atherosclerosis. Calculate the MR results of the remaining IVs after removing the IVs one by one. (A) Atherosclerosis; (B) Cerebral atherosclerosis; (C) Coronary atherosclerosis;(D) Peripheral atherosclerosis.


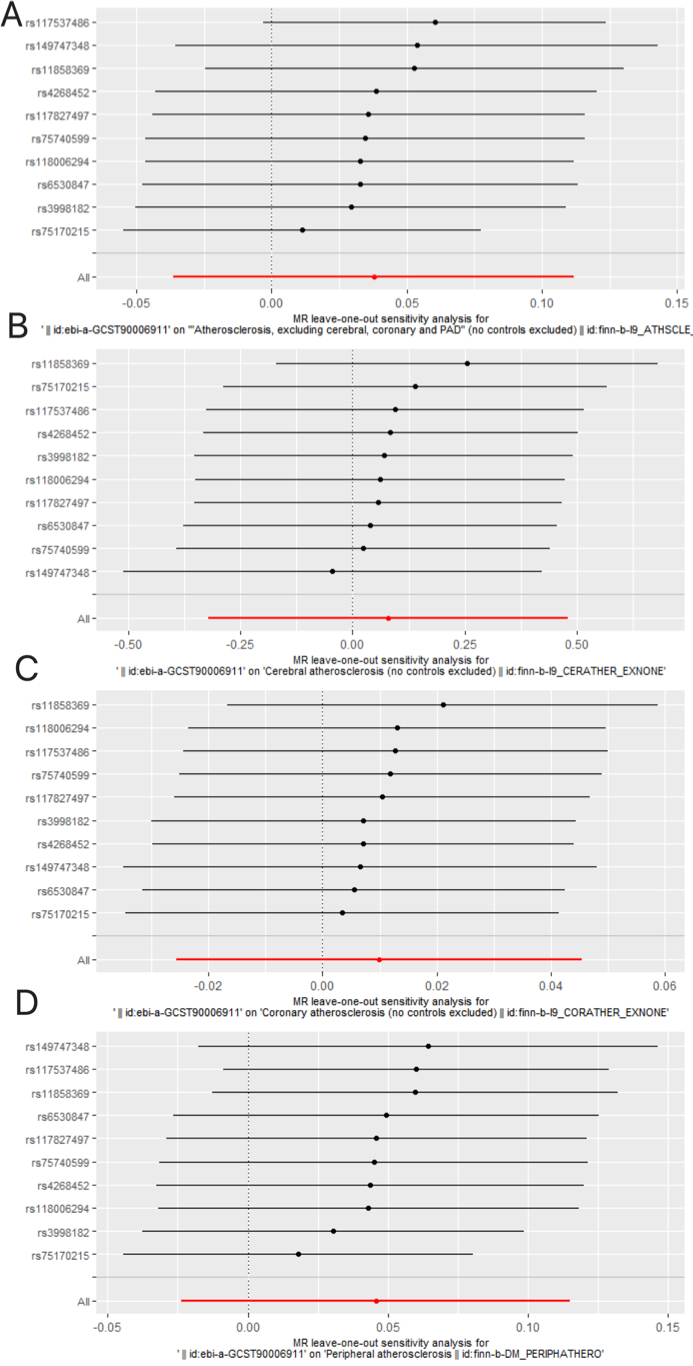


Supplementary Figure 7. Leave one out of sensitivity test of helicobacter pylori CagA antibody on atherosclerosis.(A) Atherosclerosis; (B) Cerebral atherosclerosis; (C) Coronary atherosclerosis;(D) Peripheral atherosclerosis.


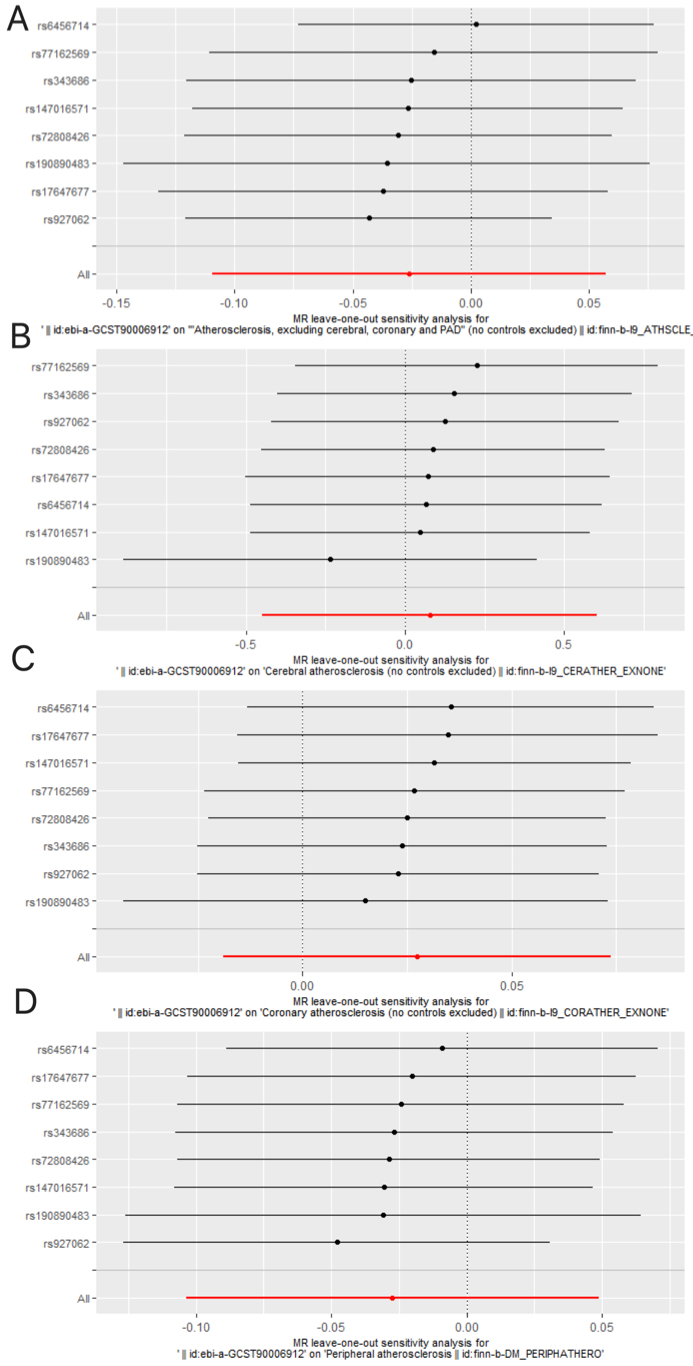


Supplementary Figure 8. Leave one out of sensitivity test of helicobacter pylori Catalase antibody on atherosclerosis. (A) Atherosclerosis; (B) Cerebral atherosclerosis; (C) Coronary atherosclerosis;(D) Peripheral atherosclerosis.


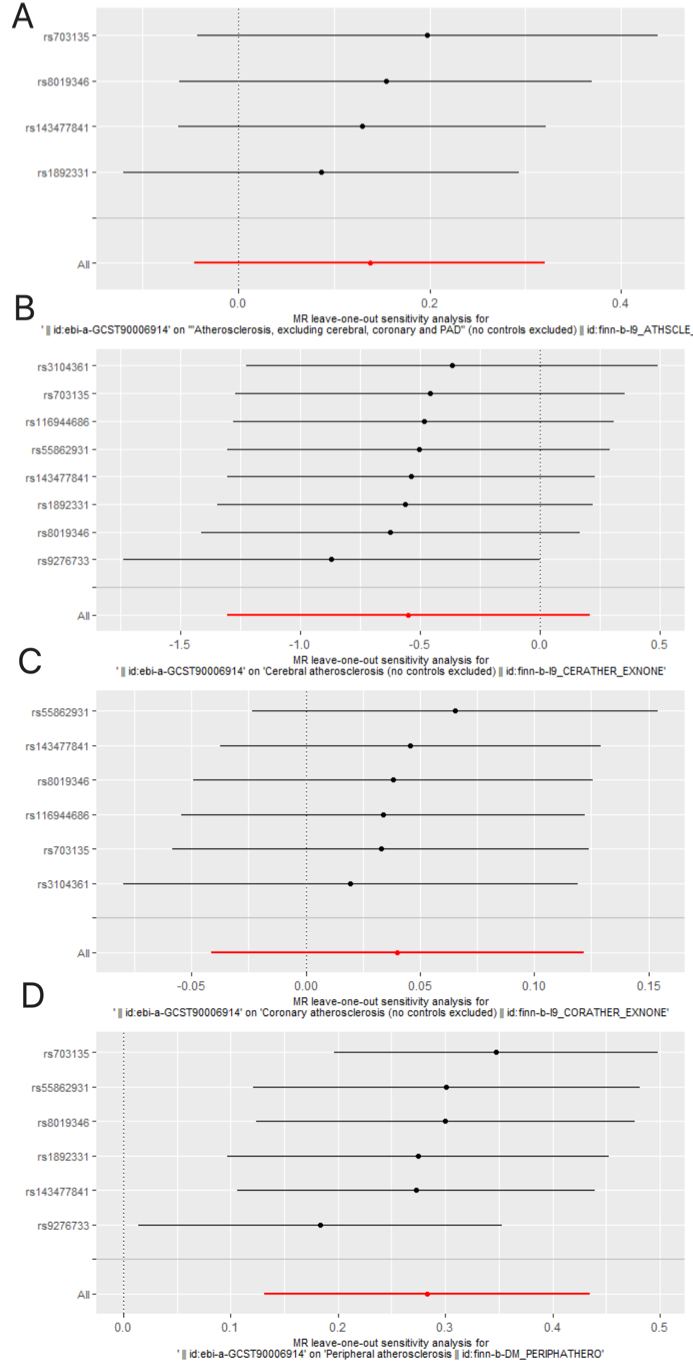


Supplementary Figure 9. Leave one out of sensitivity test of helicobacter pylori OMP antibody on atherosclerosis. Calculate the MR results of the remaining IVs after removing the IVs one by one. (A) Atherosclerosis; (B) Cerebral atherosclerosis; (C) Coronary atherosclerosis;(D) Peripheral atherosclerosis.


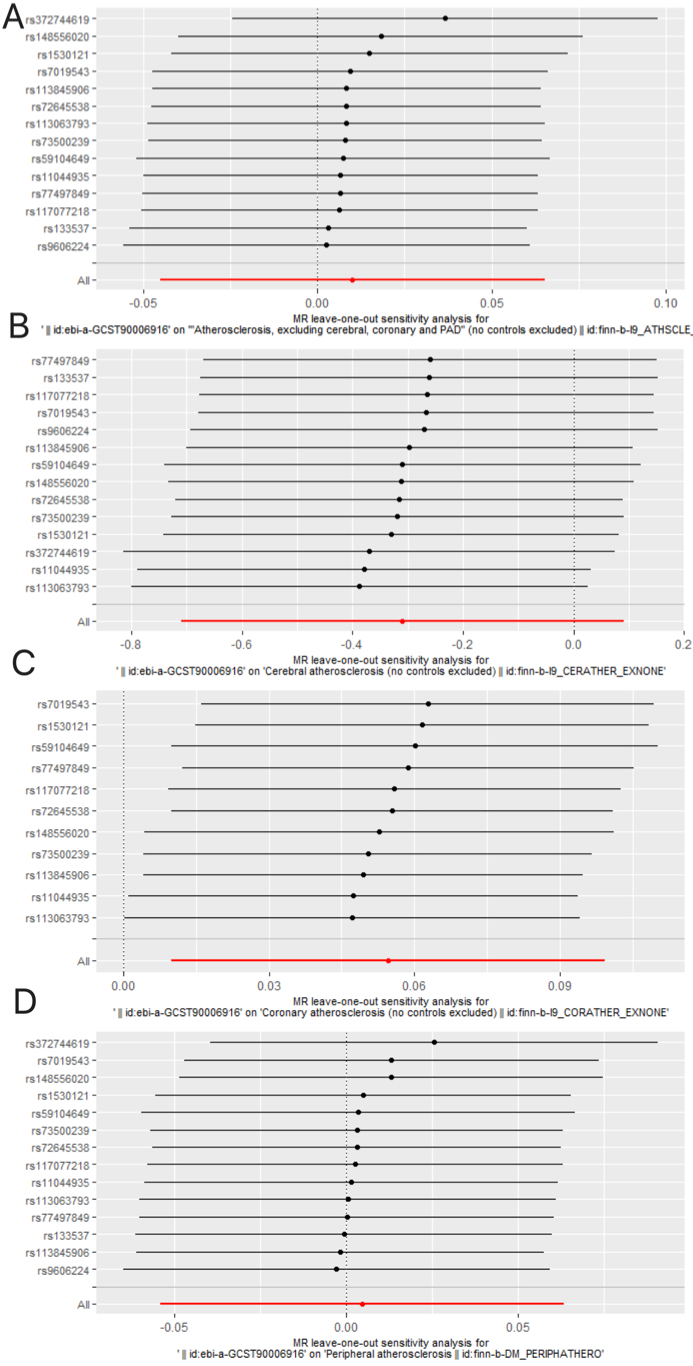


Supplementary Figure 10. Leave one out of sensitivity test of helicobacter pylori VacA antibody on atherosclerosis. (A) Atherosclerosis; (B) Cerebral atherosclerosis; (C) Coronary atherosclerosis;(D) Peripheral atherosclerosis.
